# Strengthening general practice/family medicine in Europe—advice from professionals from 30 European countries

**DOI:** 10.1186/s12875-017-0653-x

**Published:** 2017-08-22

**Authors:** Natalia Zarbailov, Stefan Wilm, Howard Tandeter, Francesco Carelli, Mette Brekke

**Affiliations:** 1 Family Medicine Department, State University of Medicine and Pharmacy “Nicolae Testemitanu”, Bd. Stefan cel Mare 165, MD-2004 Chisinau, Republic of Moldova; 2 Institute of General Practice, Heinrich-Heine-University, Werdener Str. 4, D-40227 Düsseldorf, Germany; 3 Department of Family Medicine and Siaal research center for family medicine and primary care, Ben-Gurion University of the Negev, Beer-Sheva, Israel; 4 University of Milan, Francesco Sforza 35, Milan, Italy; 5 Department of General Practice, Institute of Health and Society, University of Oslo, P.O. Box 1132 018, Blindern, Norway

**Keywords:** General practice, Family medicine, Europe, EURACT, Quality improvement, Mixed methods

## Abstract

**Background:**

Substantial variations are still to be found in the strength of general practice/family medicine (GP/FM) across Europe regarding governance, workforce competence and performance, as well as academic development and position. Governments are encouraged by the WHO to secure high quality primary health care to their population, a necessity for reaching the goal “Health for all”. The present study aimed at investigating the opinions of council members of the European Academy of Teachers in General Practice (EURACT) on necessary actions to strengthen the position of GP/FM in their country.

**Methods:**

The study used a mixed methods exploratory sequential design. EURACT representatives from 32 European countries first participated in brain-storming on how to strengthen GP/FM in Europe. Later, representatives from 37 countries were asked to individually score the relevance of the proposed actions for their country on a 9-point Likert scale. They were also asked to evaluate the status of GP/FM in their country on four dimensions.

**Results:**

Respondents from 30 European countries returned complete questionnaires. To build and secure GP/FM as an academic discipline comprising teaching and research was seen as essential, regardless the present status of GP/FM in the respective country. To build GP/FM as a specialty on the same level as other specialties was seen as important in countries where GP/FM held a strong or medium strong position. The importance of common learning objectives and a defined bibliography were stated by respondents from countries where GP/FM presently has a weak position.

**Conclusions:**

In order to strengthen GP/FM throughout Europe, EURACT and other professional organizations must establish common goals and share expertise between countries. To influence decision makers through information on cost-effectiveness of a GP/FM-based health care system is also important.

## Background

Primary Health Care was identified as central to achieve the goal “Health for all” launched by WHO at the Alma Ata conference in 1978 [1]. Thirty years later WHO encouraged all countries to orient their health care systems towards a strengthened primary health care [2], with general practice/family medicine (GP/FM) as core of primary medical care. There is increasing evidence that a strong primary health care system is more likely to provide better population health, more equity in health throughout the population, and better use of economic resources, compared to systems oriented towards specialty care [3–5].

In the “WHO global strategy on integrated people-centered health services 2016–2026” [6] building strong primary care-based systems is highlighted as a necessity. The document states that strong primary care services are essential for reaching the entire population and guaranteeing universal access to services. This includes ensuring adequate funding, appropriate training, and connections to other services and sectors [6]. The strength of GP/FM in a country can be assessed by outcome measures covering several dimensions: governance, economic conditions, workforce development, accessibility, comprehensiveness, continuity, and coordination of care [7]. How GP/FM is defined as a clinical specialty, if and how it is taught on undergraduate level in medical schools, as well as its position in research, are also important markers of its strength and position [8, 9].

Substantial variations are still to be found in the strength of GP/FM across Europe – regarding governance, workforce development and performance [7], as well as academic development and position [10]. It is still possible to graduate from a European medical school without having learned the principles of GP/FM and without any clinical teaching in a GP's office [10].

Each country is responsible for improving development of primary care and securing and sustaining its position. European professional organizations should facilitate such an improvement, like the European Academy of Teachers in General Practice/Family Medicine (EURACT) [11] did by its statement to encourage all medical faculties and departments across Europe to implement teaching and training programs of GP/FM into their curriculum [9] and to develop a minimal core curriculum [12]. EURACT was launched in March, 1992 as the European educational wing and network organization of World Organization of Family Doctors (WONCA) [13]. EURACT has over 800 members in nearly 40 countries. Its overall aim is to foster and maintain high standards of care in European GP/FM by promoting GP/FM as a discipline by learning and teaching. Council members are elected among the EURACT members in each country. The present study aimed at investigating these council members’ opinions on necessary actions to strengthen the position of GP/FM in their country – in order to get a European overview.

## Methods

### Study design

The study used a mixed methods exploratory sequential design [14]. This design begins with a qualitative data collection and analysis phase, which builds to the subsequent quantitative phase. Participants first launched possible actions needed to strengthen GP/FM in Europe through brain-storming. Later, they individually scored the relevance of the proposed actions for their country on a questionnaire.

### Participants

The participants were GPs from 32 (brain-storming process) or 30 (questionnaire study) European countries plus Israel, who at the time of the study were elected council members of EURACT [11]. EURACT council members are elected by and out of all EURACT members in their respective country. As EURACT is an organization dedicated to teaching in GP/FM, their members are mainly experienced academic GPs involved in undergraduate teaching, vocational training and/or continuous medical education. Most – but not all – also work part time in clinical practice and most have research experience. To be elected as a EURACT council member requires that the person stands a trusted and respected position among academic GPs in his/her country.

The election period is 3 years, but may be extended. At present, EURACT has all together 38 council members from 37 European countries plus Israel. Out of these, 15 are men and 23 are women, and most are between 40 and 65 years of age. These council members represent countries with a great variety of GP/FM’s academic development and position as well as in the profession’s strength in the health care system.

### Data collection

Data collection consisted of a brain-storming process and a subsequent questionnaire study and took place from October 2013 until December 2014. The EURACT council meets twice a year on different locations. During a meeting in October 2013 in Tirana, Albania [15], the council members were asked to brain-storm in two consecutive sessions around the question: “Which activities to strengthen general practice/family medicine are relevant for European countries?” Representatives from 32 countries out of the 37 member countries (by Oct 2013) were present at the meeting and participated in the brain-storming. The research question, the design of the study and the sessions were planned in advance by the authors, whereas the other council members were not informed about the content of the study beforehand. The sessions took place in plenary and were conducted by two of the authors (HT, NZ).

All council members were active in the process. The facilitating question was “Which activities to strengthen general practice/ family medicine are relevant for *your* country?” The answers of the participants were protocoled verbatim and generated a list of more than 60 possible actions.

The two facilitators revised the list and agreed upon doublets, which were deleted. Otherwise, the items were kept in the original version proposed during the session. This resulted in a list of 50 items.

This list of 50 possible actions to strengthen GP/FM in Europe was subsequently sent to the 37 EURACT representatives by e-mail. They were asked to score the importance of the individual items for their country by means of a 9-poins Likert scale from 1 (strongly disagree that this action is important to strengthen GP/FM in my country) to 9 (strongly agree), with 5 being neutral. Main outcome was the panelists’ evaluation of each statement as scored on the Likert scale. Respondents were also asked to evaluate the status of GP/FM in their country on four dimensions: At policy/governance level, at academic/university level, at population acceptance level and the importance for health care system progress – by means of the scores low, medium and high. Based on these scores, the countries were grouped in “high level”, “medium level” and “low level” countries.

### Statistics

The scores from the questionnaire study were plotted into SPSS version 22. For each criterion, mean scores with standard deviation (SD) was calculated. Agreement on importance for a statement was obtained if the mean score minus one SD exceeded 5 (called “final score”). Subgroup analyses were carried out for “high level”, “medium level” and “low level” countries.

## Results

Out of the 37 EURACT representatives asked to score the importance of the 50 possible actions needed to strengthen GP/FM, 30 returned complete questionnaires.

The 50 items with mean score (min – max) on a 9-point Likert scale and SD, as well as the “final score” are shown in Table 1 in decreasing order of importance.

**Table 1:** Possible actions to strengthen general practice/family medicine (GP/FM) in Europe – scores on a 9-point Likert scale (1 = strongly disagree on importance, 9 = strongly agree, *n* = 30 respondents)

Possible action to strengthen GP/FM in Europe	Mean score	Min - max	SD	Final score
To keep family doctors as leaders of GP/FM university departments	8.36	5–9	0.99	7.37
To build the same level of specialty as other disciplines	8.29	3–9	1.54	6.75
To train academic teaching staff, to run “Training of Trainers”	7.96	5–9	1.35	6.61
To create a network of GP/FM university departments	7.89	5–9	1.29	6.60
To develop staff and invest in academic development, motivate young staff to build an academic career	7.66	5–9	1.08	6.58
To have strong leaders	7.89	4–9	1.32	6.57
To remind that to prepare doctors for primary care is a priority for Ministry of Health and universities	7.82	4–9	1.36	6.46
To look at what specific help individual countries need	7.54	5–9	1.31	6.24
To keep working and proving yourself	7.68	4–9	1.43	6.18
To invest in research and common research projects	7.60	3–9	1.45	6.15
To know existing statement papers and create a common list of documents, bibliography and selected readings	7.57	4–9	1.45	6.12
To build a strong scientific society	7.83	1–9	1.74	6.09
To introduce a mentor system with one to one teaching and create a list of tutors for teaching	7.39	3–9	1.52	5.87
To empower EURACT, make transparency and visibility	7.32	4–9	1.49	5.83
To list the competencies for undergraduate level	7.39	4–9	1.69	5.70
To establish common learning objectives	7.25	1–9	1.65	5.60
To create a link between academics and policy makers	7.23	4–9	1.63	5.60
To collaborate with other organizations	7.10	3–9	1.61	5.49
To find a “right person” to get in touch with Minister of Health, rector, etc.	7.11	3–9	1.71	5.40
To use public opinion for political pressure	7.10	4–9	1.71	5.39
To find what are the patient’s needs and make patients our partners	6.96	3–9	1.44	5.32
To stress the cost- effectiveness	7.07	1–9	1.84	5.23
To involve media to show leaders and/or popular persons	6.75	4–9	1.53	5.22
To make GP/FM attractive - “sexy”	6.89	4–9	1.69	5.20
To introduce a special course on GP/FM for first year students and early clinical exposure	7.00	3–9	1.87	5.13
To work on good examples	7.36	1–9	2.20	5.16
To introduce certification for GP/FM educators	6.79	1–9	1.83	4.96
To use experts’ help and invite experienced colleagues from other countries to establish a GP/FM university department	7.04	1–9	2.12	4.92
To provide databases and web resources	6.75	3–9	1.88	4.87
To learn from those who succeed to establish a GP/FM University Department	6.96	1–9	2.15	4.81
To present goals at national conferences	6.83	1–9	2.02	4.81
To use modern technology	6.71	1–9	1.94	4.77
To use help of whole Europe	6.71	1–9	1.97	4.69
To show documents to the governments and media	6.25	4–9	1.58	4.67
To prepare a position paper to define university curriculum and include GP/FM in university curriculum	7.10	1–9	2.52	4.58
To look at the common GP/FM curriculum in the country	6.54	3–9	2.01	4.53
To write an open letter, describe who you are, what are your goals and objectives, what is done already, what are your future aspirations, offer yourself as an expert, volunteer to help	6.32	2–9	1.83	4.49
To organize educational research on “the best curriculum”	6.68	1–9	2.21	4.47
To know what others do, find common points with specialists and look for specialists’ allies and help, share and collaborate with them	6.11	4–9	1.69	4.42
To exchange teachers	6.43	1–9	2.19	4.11
To post an English version of the curriculum on the university website	6.29	1–9	2.39	3.90
To implement a GP/FM approach in other subjects/disciplines	6.21	1–9	2.42	3.88
To improve European legislation	5.80	1–9	2.20	3.60
“Touch the hearts”/ show movies	5.86	1–9	2.42	3.44
To define what is GP/FM	5.43	1–9	2.69	2.74
To organize GP/FM training for other specialists	4.04	1–9	2.69	1.35
To push deans to specialize in GP/FM	3.54	1–8	2.50	1.04
To organize workshops on cardiology, hematology, neurology etc. for GPs	3.46	1–9	2.29	.67
To stop fighting	3.54	1–9	3.01	.53
To start fighting	3.43	1–9	3.02	.41

Final score = mean score – 1 SD

For 26 out of the 50 items, mean score minus one SD (“final score”) exceeded 5. These 26 items were grouped by the authors into the following five categories: Actions to strengthen GP/FM as an academic discipline, Actions to develop GP/FM as a specialty, Actions to influence decision makers, Organizational work, and Personal work. The five categories are shown in Table 2 with the item with the highest “final score” on top.

**Table 2:** Important actions to strengthen general practice/family medicine (GP/FM) in Europe (Final score = mean score minus 1 SD on a 9-point Likert scale, 1 = strongly disagree on importance, 9 = strongly agree, *n* = 30 respondents)

	Mean score	SD	Final score
Actions to strengthen GP/FM as an academic discipline			
To keep family doctors as leaders of GP/FM departments – the teaching process and decision making	8.36	0.99	7.37
To train academic staff, to run “Training of Trainers”	7.96	1.35	6.61
To create a network of GP/FM university departments	7.89	1.29	6.60
To develop staff and invest in academic development, motivate young staff to build an academic career	7.66	1.08	6.58
To invest in research and common research projects	7.60	1.45	6.15
To build a strong scientific society	7.83	1.74	6.09
Actions to develop GP/FM as a specialty			
To build the same level of specialty as other disciplines	8.29	1.54	6.75
To know existing statement papers and create a common list of documents, bibliography and selected readings	7.57	1.45	6.12
To introduce a mentor system with one to one teaching and create a list of tutors for teaching	7.39	1.52	5.87
To list competencies for undergraduate level	7.39	1.69	5.70
To establish common learning objectives	7.25	1.65	5.60
To make GP/FM attractive - “sexy”	6.89	1.69	5.20
To introduce a special course on GP/FM for first year students and early clinical exposure	7.00	1.87	5.13
Actions to influence decision makers			
To have strong leaders	7.89	1.32	6.57
To remind that to prepare doctors for primary care is a priority for Ministry of Health and universities	7.82	1.36	6.46
To create a link between academics and policy makers	7.23	1.63	5.60
To find a “right person” to get in touch with Minister of Health, rector, etc. – direct your efforts to decision makers	7.11	1.71	5.40
To use public opinion for political pressure	7.10	1.71	5.39
To find what are the patient’s needs and make patients our partners	6.96	1.44	5.32
To stress the cost- effectiveness	7.07	1.84	5.23
To involve media to show leaders and/or popular persons	6.75	1.53	5.22
Organizational work			
To look at what specific help individual countries need	7.54	1.31	6.24
To empower EURACT, make transparency and visibility	7.32	1.49	5.83
To collaborate with other organizations	7.10	1.61	5.49
Personal work			
To keep working and proving yourself	7.68	1.43	6.18
To work on good examples	7.36	2.20	5.16

Importance = Final score > 5.0

The respondents’ ratings of GP/FM development in their country on policy/governance level, on academic/university level, on population acceptance level and regarding importance for health care system progress are shown in Table 3. Thirteen out of the 30 countries obtained a total high development score, 11 a medium score, whereas six countries scored low.

**Table 3:** Respondents’ rating of general practice/family medicine development in their country (*n* = 30)

Country	Policy/Governance Low = 1 Medium = 2 High = 3	Academic/University Low = 1 Medium = 2 High = 3	Population acceptance Low = 1 Medium = 2 High = 3	Health care system progress Low = 1 Medium = 2 High = 3	General Score Low = 4–6 Medium = 7–9 High = 10–12
High(*n* = 13)					
Netherlands	3	3	3	3	12
Israel	3	3	3	3	12
United Kingdom	3	3	3	3	12
Denmark	3	3	3	2	11
Estonia	2	3	3	3	11
Turkey	2	3	3	3	11
Bosnia-Herzegovina	2	2	3	3	10
Germany	3	2	3	2	10
Lithuania	1	3	3	3	10
Malta	2	3	3	2	10
Norway	2	3	3	2	10
Portugal	2	3	2	3	10
Romania	2	2	3	3	10
Medium (*n* = 11)					
Croatia	2	3	2	2	9
Czeck Rep.	2	3	2	2	9
Finland	2	3	2	2	9
Slovenia	2	3	2	2	9
Sweden	3	2	2	2	9
Italy	1	1	3	3	8
Montenegro	2	2	2	2	8
Poland	1	2	3	2	8
Slovakia	2	2	2	2	8
Spain	2	1	3	2	8
Bulgaria	2	2	1	2	7
Low (*n* = 6)					
Austria	1	1	2	2	6
Georgia	1	2	1	2	6
Macedonia	1	2	1	2	6
Moldova	2	1	1	2	6
Russia	1	2	2	1	6
Serbia	1	1	2	2	6

Based upon subgroup analyses on the 50 items for the countries labelled as high, medium and low level countries (data not shown in Table), the ten items with highest score in each group were identified (Table 4).

**Table 4:** Top ten items for countries with high, medium and low rating of general practice/family medicine (GP/FM) status. Scores on a 9-point Likert scale (mean score minus 1SD)

Countries with high rating (*n* = 13)		
Rank	Item	Score
1	To develop staff and invest in academic development, motivate young staff to build an academic career	7.22
2	To build the same level of specialty as other disciplines	7.15
3	To have strong leaders	7.13
4	To keep family doctors as leaders of GP/FM departments	7.06
5	To create a link between academics and policy makers	6.76
6	To look at what specific help individual countries need	6.51
7	To collaborate with other organizations	6.30
8	To create a network of GP/FM university departments	6.27
9	To keep working and proving yourself	6.09
10	To train academic staff, to run “Training of Trainers”	6.06
Countries with medium rating (*n* = 11)		
Rank	Item	Score
1	To build a strong scientific society	7.62
2	To keep family doctors as leaders of GP/FM departments	7.56
3	To build the same level of specialty as other disciplines	7.43
4	To train academic staff, to run “Training of Trainers”	6.97
5	To have strong leaders	6.75
6	To introduce a mentor system with one to one teaching and create a list of tutors for teaching	6.75
7	To remind that to prepare doctors for primary care is a priority for Ministry of Health and universities	6.61
8	To keep working and proving yourself	6.61
9	To develop staff and invest in academic development, motivate young staff to build an academic career	6.57
10	To establish common learning objectives	6.56
Countries with low rating (*n* = 6)		
Rank	Item	Score
1	To create a network of GP/FM university departments	8.25
2	To keep family doctors as leaders of GP/FM departments	7.50
3	To establish common learning objectives	7.36
4	To post an English version of the curriculum on the university website	7.35
5	To train academic staff, to run “Training of Trainers”	7.29
6	To remind that to prepare doctors for primary care is a priority for Ministry of Health and universities	7.29
7	To use experts’ help and invite experienced colleagues from other countries to establish a GP/FM university department	7.00
8	To know existing statement papers and create a common list of documents, bibliography and selected readings	6.96
9	To introduce a mentor system with one to one teaching and create a list of tutors for teaching	6.96
10	To work on good examples	6.90

To establish and strengthen GP/FM as an academic discipline was seen as essential in all the three groups, as several of the top score items addressed this theme. To build GP/FM as a specialty on the same level as other specialties was seen as important in high- and medium score countries. The low-score countries did not prioritize this statement, whereas the importance of common learning objectives and a defined bibliography were stated – both important issues in the building of a specialty. Low score countries valued “To use experts’ help and invite experienced colleagues from other countries to establish a GP/FM university department”, while high score countries agreed on the importance of “To look at what specific help individual countries need”. Actions to inform and influence policy makers about the importance of GP/FM were listed by all three groups, as were “To keep working and proving yourself” and “To work on good examples”.

## Discussion

There was substantial agreement on actions needed to strengthen GP/FM among EURACT council members from 30 European countries. The proposed actions were oriented towards two main themes: First, to improve the quality of GP/FM as a profession, and second, to optimize the circumstances under which the GP’s work is carried out. Actions to improve quality focused on two main tasks: to build and secure GP/FM as an academic discipline comprising teaching and research and to develop GP/FM as a clinical specialty.

General practice is probably the only health care setting where patients can freely address their worries and present several, unrelated and often complex problems [16]. Ageing and increasingly multi-morbid populations demand that multiple problems have to be dealt with in consultations [17]. To deal repeatedly with such complaints within brief encounters and to translate them into medically sound actions is said to be the most challenging intellectual exercise in clinical medicine [18]. Thus, to work as a GP demands a specific type of competence, more comprehensive and different from traditional knowledge regarding diseases and their treatment [5, 19]. This has implications for medical education, both undergraduate, and in particular for GPs' vocational training.

The quality level of GP/FM services still varies between European countries [20]. An example is Norway, where until now there has been no mandatory postgraduate training for GPs at all. In our study, respondents saw actions to develop GP as a specialty as highly important – and “to build the same level of specialty as other disciplines” got top score (Table 2).

Actions to strengthen undergraduate GP/FM teaching were also agreed upon as important, like “to list competencies for undergraduate level”, and “to introduce a special course on GP/FM for first year students and early clinical exposure”. It is still possible to graduate from a European medical school without having been exposed to a GP/FM curriculum [10]. EURACT will continue to launch efforts so that all medical students will complete a GP/FM curriculum [12], as well as a GP/FM clerkship [9].

High quality GP/FM undergraduate and postgraduate teaching requires a correspondingly high academic level of the profession, as seen as essential by our study participants. This also comprises “investment in research and common research projects” (Table 2). It is widely accepted that knowledge borrowed or adapted from other specialties is insufficient to create an evidence base for the comprehensive medical care that takes place in general practice [8, 21, 22]. Investment in and level of GP/FM research, however, varies among European countries [23, 24], and generally still has potential for improvement [25].

The second main theme proposed by our respondents as essential was to optimize the structural conditions for GP/FM. Strong primary care does not emerge spontaneously, but requires facilitating political and economic circumstances. In a recent paper Schäfer et al. investigated changes in the breadth of services provided by GPs in 28 European countries from 1993 to 2012 and possible explanations for these changes [20]. They found that conditions on the national level were associated with changes in the service profile of GPs. In countries with stronger growth of health care expenditures the service profiles had expanded. This reality seems clear to our informants, as they propose several actions to influence national health policy: through strong leaders and academia, through media and public opinion, as well as with the help of patients (Table 2). “To stress the cost- effectiveness” is another action proposed by our respondents to help influence health policy in their country. The favourable cost-effectiveness of a GP/FM oriented health care system versus one based upon specialists and hospitals has been clearly stated [2–4].

While stronger primary care could be seen as a common solution when facing limited resources, European countries have responded differently to these challenges. WHO defines health policy as “decisions, plans and actions that are undertaken to achieve specific health goals within a society” [26]. Mackenbach et al. investigated variations in health policy regarding ten areas of preventive tasks in 43 European countries and found striking variations between countries in process and outcome indicators of health policy [27]. They concluded that substantial health gains can be achieved if all countries would follow best practice, but that this depends on both the “will” and the “means” of governments to implement such a health policy.

Our study participants were asked to rank the position of GP/FM in their country on several dimensions, leading to a label of “high”, “medium” or “low” (Table 3). Several studies have investigated the performance of primary care systems in Europe in terms of quality, equity and costs [7, 28–32]. We could have used data from these studies for our subgroup analyses – although some were not published at the time the present study was planned. However, their labeling only showed minor deviations compared to the ranking done by the EURACT representatives [7, 30] which we therefore view as reliable for the purpose of this study. There were only minor variations between the three groups in what was considered important, as shown in Table 4 (even if the study is too small to allow for reliable statistical analysis on this matter). Countries where GP/FM has a high position were open to “look at what specific help individual countries need”, whereas the “low position” countries expressed the wish to “use experts’ help and invite experienced colleagues from other countries to establish a GP/FM university department”. As Seifert et al. point out regarding Central- and Eastern European countries: There is a need for continuous exchange of expertise between countries – e.g. to promote and secure academic infrastructure and CME [23].

The strength of our study was that representatives from 32 countries participated in creating the list of the 50 possible actions, securing that a multitude of opinions were exposed, and that 30 representatives subsequently carried out the scoring. One limitation of the study was that we were not able to obtain questionnaire data from some countries: Albania, Belgium, Greece, Hungary, Ireland, Latvia and Switzerland, due to non-response. A further limitation was the subjective character of the responses, coming from just one person representing one country, even if the participants stand a trusted position in their respective countries.

## Conclusion

EURACT and other professional organizations have a responsibility to promote a strong and high quality GP/FM throughout Europe. This implies to establish common goals and to share expertise between countries. The aims are that every European medical school should have an undergraduate GP/FM curriculum and a residency. Vocational training should be accessible and mandatory. The academic position of GP/FM should be strengthened, and research should be facilitated. Working conditions must be improved – so decision makers must be influenced, by information on cost-effectiveness and potential health gains through a health care system based on strong GP/FM.

## Abbreviations

EURACT: European Academy of Teachers in General Practice/Family Medicine
GP/FM: General practice/family medicine
SD: Standard deviation
WHO: World Health Organization
WONCA: World Organization of Family Doctors
